# A critical review of Chinese vaccine enterprises in the global aid market: evolution, drivers, and structural constraints

**DOI:** 10.3389/fpubh.2025.1692140

**Published:** 2025-12-05

**Authors:** Mengli Ding

**Affiliations:** School of Politics and Public Administration, Soochow University, Suzhou, China

**Keywords:** global health governance, global vaccine aid market, Chinese vaccine enterprises, equitable vaccine access, COVID-19

## Abstract

The Chinese government has assumed an increasingly prominent role in global health governance in recent years, yet the engagement of Chinese enterprises remains underexplored. Existing studies have largely focused on Beijing’s major initiatives during the COVID-19 pandemic, overlooking the evolving role of Chinese enterprises as non-state actors. This article provides a comprehensive review of the participation of Chinese vaccine companies in the global aid market, with particular attention to their changing roles, underlying motivations, and persistent challenges as latecomers from the Global South. Drawing on around 80 industry and policy documents, publicly available datasets, and 10 semi-structured interviews with industry and government stakeholders, this study uses documentary synthesis and thematic analysis to show that COVID-19 marked a critical turning point, accelerating Chinese vaccine manufacturers’ international engagement while exposing persistent structural barriers. Unlike earlier studies, this review shows that Chinese vaccine enterprises’ engagement in the global aid market is shaped not only by strategic market ambitions and alignment with national public health priorities, but also by enduring structural constraints such as geopolitical tensions, institutional mistrust, talent shortages, and reputational vulnerabilities. By situating Chinese enterprises within broader debates on non-state actors in global health, the study advances existing literature and offers policy-relevant insights to strengthen their capacity and influence in promoting equitable vaccine access.

## 1 Introduction

In recent years, the Chinese government has assumed an increasingly prominent role in global health governance, shifting from a primarily recipient position to that of an active agenda-setter with its own health initiatives and conceptual frameworks. A milestone moment came in June 2016, when President Xi Jinping, during his visit to Uzbekistan, first proposed the idea of “jointly building a Health Silk Road,” framing health cooperation as an integral component of the Belt and Road Initiative (BRI) (1). The Health Silk Road (HSR) has since been described as the health dimension of the BRI, emphasizing bilateral and multilateral health cooperation, public health infrastructure development, and enhanced capacities for pandemic preparedness and response (2). This vision was further elevated at the Global Health Summit on May 21, 2021, where Xi emphasized China’s unwavering commitment to international cooperation against COVID-19 and articulated the concept of a “a community of health for mankind,” extending the broader “community of shared future for mankind” into the field of health governance (3). These high-level policy signals have drawn increasing scholarly attention.

In reviewing the past decade of literature on China’s participation in global health governance, we conducted a systematic search across authoritative academic databases. A combination of keywords such as “China,” “global health governance,” “Health Silk Road,” “vaccine diplomacy,” was applied. To enhance transparency and reproducibility, this review adhered to the Preferred Reporting Items for Systematic Reviews and Meta-Analyses (PRISMA) framework, adapted for policy-oriented scoping reviews (PRISMA-ScR). A systematic literature search was performed across two major academic databases—Web of Science and PubMed—covering the period from January 2015 to May 2025. To address potential database coverage gaps, Google Scholar was used as a supplementary source. Comprehensive search strings incorporating Boolean operators and truncations were applied (see Supplementary Table S1 for the full strategies). All retrieved records were exported, de-duplicated, and screened through a two-stage process: (i) title and abstract screening, followed by (ii) full-text assessment.

**Figure 1:**
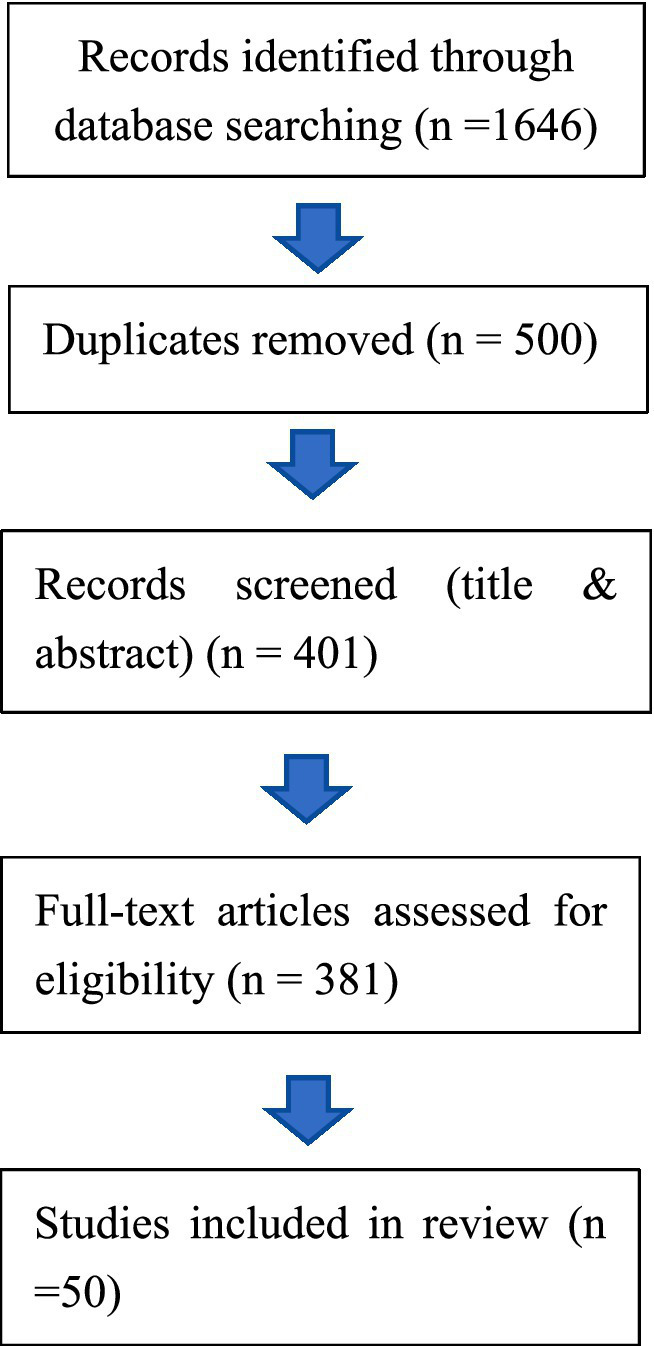
Literature screening and selection process based on the PRISMA-ScR framework. Source: Authors’ elaboration based on the PRISMA-ScR framework.

Studies were included if they explicitly examined China’s role, strategies, or influence in global health governance or vaccine-related international cooperation. Records were excluded if they (i) focused primarily on domestic health reforms in China, (ii) discussed global health governance without treating China as a central analytical subject, or (iii) consisted of editorials, policy briefs, news reports, or purely descriptive documents lacking analytical depth. Figure 1 presents the PRISMA flow diagram summarizing the screening process and document counts at each stage. Given that this review seeks to synthesize policy-relevant insights rather than assess intervention effectiveness, no formal quality appraisal was conducted. This decision aligns with established guidance for scoping and policy reviews, in which the heterogeneity of evidence types (academic articles, institutional reports, and grey literature) limits the applicability of standardized quality metrics.

Building on the systematic review described above, the following section synthesizes key insights from the existing literature on China’s global health engagement. The existing scholarship has predominantly examined China’s state-led global health diplomacy—such as its promotion of the HSR—and the underlying motives and broader political and normative implications of its health cooperation agenda (4–6). A significant body of work further analyzes China’s practices during the COVID-19 pandemic, including a mix of bilateral and multilateral strategies (7), its vaccine diplomacy and its implications (8, 9), and the adaptation of the HSR to advance pandemic cooperation while mitigating external criticisms (10, 11).

While this scholarship has enriched our understanding of China’s engagement in global health as a state actor, it has largely overlooked the evolving role of Chinese enterprises as non-state actors. In particular, limited attention has been paid to the participation of Chinese vaccine manufacturers in the global vaccine aid market—a distinct segment of the international vaccine trade that involves public sector intervention and is characterized by procurement through multilateral organizations such as Gavi, the Vaccine Alliance (GAVI), and the United Nations International Children’s Emergency Fund (UNICEF). This market is driven by aid-based principles aimed at improving vaccine access for developing countries, but it also retains market-like dynamics in terms of procurement processes and distribution. This review seeks to fill that gap by systematically examining the role of Chinese vaccine manufacturers in the global vaccine aid market over the past decade. Specifically, it aims to (1) identify the drivers and motivations behind their participation, (2) analyze the external and internal constraints they face as late entrants, and (3) assess the broader implications of their engagement for China’s global health governance and for the provision of vaccines as global public goods. By delineating these objectives, the review establishes both its analytical scope and scholarly contribution by bridging state-centered analyses of China’s global health diplomacy with research on non-state actors in global health governance. Building on this analytical foundation, the review further underscores the practical importance of developing adaptive, phased strategies within multilateral institutions to more effectively mitigate resource and institutional asymmetries and to enhance the feasibility of equitable vaccine access in practice.

Methodologically, this study adopts a qualitative research design integrating documentary analysis, official data review, and semi-structured interviews. Documentary materials include industry reports, policy papers, authoritative media reports, and peer-reviewed studies on the internationalization of Chinese vaccine enterprises and their engagement in global aid procurement mechanisms. Official statements and datasets were obtained from the Chinese government—including the National Health Commission—as well as from the official websites of international health institutions such as the World Health Organization (WHO), GAVI, and UNICEF. In addition, news reports from both Chinese and international media outlets—such as Xinhua News Agency and the BBC—were consulted to capture public narratives from multiple perspectives. To ensure data reliability, all sources were cross-verified through independent references and validated by confirming their institutional, journalistic, or peer-reviewed credibility.

To fill existing empirical gaps and capture firsthand perspectives, this study draws on 10 semi-structured interviews and informal consultations conducted between January 2020 and May 2025 with key stakeholders involved in China’s engagement in the global vaccine aid market. Respondents were identified through purposive and snowball sampling, targeting individuals directly involved in policy design, partnership coordination, or enterprise-level implementation. Recruitment relied on professional and institutional networks and was complemented by follow-up exchanges at policy dialogues and academic events.

The interviewees included senior officials from Chinese government agencies overseeing global health or development cooperation, representatives of international organizations such as Coalition for Epidemic Preparedness Innovations (CEPI), GAVI, and the Bill & Melinda Gates Foundation, and executives from leading Chinese vaccine manufacturers. Participants were based in Beijing, Shanghai, and Geneva, reflecting diverse institutional and regional perspectives.

All participants were informed of the study’s objectives, confidentiality arrangements, and voluntary participation principles, with verbal or written consent obtained prior to information use. Interview notes and transcripts were thematically analyzed using a constant comparison approach, and inter-coder consistency was ensured through joint coding and discussion. To enhance analytical validity, interview insights were triangulated with policy documents and secondary literature, while additional exchanges during the Medicine and International Relations Conference (October 2020) further contextualized China’s pharmaceutical internationalization under the broader Health Silk Road framework.

To address empirical gaps, 10 semi-structured online interviews were conducted between January 2020 and May 2025 with key stakeholders involved in China’s participation in the global aid vaccine market. Interviewees included senior officials from relevant government departments, representatives from international organizations such as the CEPI, GAVI, and the Gates Foundation, and executives from leading Chinese vaccine enterprises. Selection prioritized individuals directly engaged in policy design, partnership coordination, or enterprise-level implementation, while those without substantive involvement were excluded. Additionally, participation in the academic conference Medicine and International Relations (October 2020) provided valuable insights through informal exchanges with influential scholars on the international expansion of China’s pharmaceutical industry and its significance within the broader Health Silk Road framework. The triangulation of interview data, documentary analysis, and validated secondary sources strengthens the methodological rigor and credibility of the findings.

This paper makes three contributions. First, it conceptualizes the global vaccine aid market as analytically distinct from the self-procurement market, highlighting its unique institutional structures and operational model. Second, it provides a longitudinal analysis of Chinese vaccine manufacturers’ participation, identifying the COVID-19 pandemic as a watershed moment in their market engagement. Third, it examines the specific motivations and constraints faced by firms from the Global South, providing policy-relevant insights for international health institutions seeking to diversify vaccine supply chains and enhance equitable access in preparation for future pandemics.

## 2 Operational model of the global vaccine aid market

### 2.1 Global self-procuring vaccine market

The global vaccine market is structurally bifurcated into a self-procured segment and a donor-funded aid segment, delineated by disparities in countries’ economic capacity and domestic production infrastructure (12). Within this dual structure, wealthy small states often depend on external markets for vaccine procurement, whereas larger or industrialized high- and middle-income countries predominantly rely on domestic R&D and production to meet national immunization needs.

**Figure 2:**
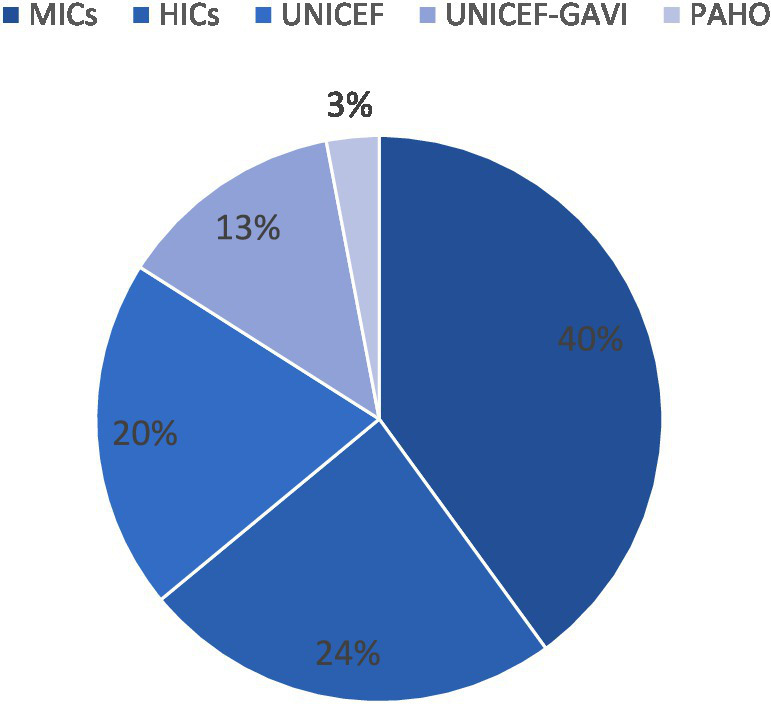
Global vaccine market procurement overview 2023. Source: World Health Organization. Global Vaccine Market Report 2024; Geneva, Switzerland, 2025. Available online at: https://www.who.int/publications/i/item/B09198 (Accessed February 3, 2025).

As shown in Figure 2, according to the WHO’s *Global Vaccine Market Report 2024*, self-procuring middle-income countries (MICs) accounted for approximately 40% of global vaccine volumes (measured in doses, calendar year 2023)—driven primarily by China, India, and Indonesia, which together contributed 27% of total global doses. High-income countries (HICs) added another 24% of total doses, underscoring the dominant role of national capacity in shaping vaccine access (13). It is noteworthy, however, that WHO statistics are derived from official country reports and may not fully capture informal or private-sector procurement. As a result, the scale of self-procuring activity may be systematically underestimated, particularly in countries with significant private-market transactions.

### 2.2 Global vaccine aid market

**Figure 3:**
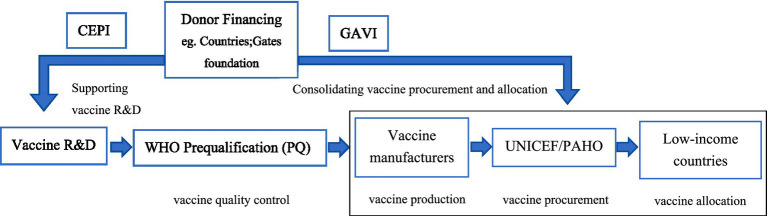
Operational model of the global vaccine aid market. Source: Authors’ schematic. Factual elements drawn from CEPI vaccine R&D support (Coalition for Epidemic Preparedness Innovations, Accessed July 22, 2025), WHO Prequalification programme (World Health Organization, Accessed July 22, 2025), UNICEF procurement data (United Nations Children’s Fund, Accessed July 22, 2025), GAVI financing and delivery (Gavi, the Vaccine Alliance, Accessed July 22, 2025), and PAHO Revolving Fund operations (Pan American Health Organization, Accessed 22 July 2025).

The global vaccine aid market refers to the segment of the international vaccine trade that is specifically designed to address vaccine access issues in low-income countries, where financial, technical, and logistical challenges hinder effective vaccine procurement through commercial channels. Unlike the self-procuring market, where countries or organizations independently purchase vaccines, the aid market is defined by public sector involvement, multilateral cooperation, and donor financing. This market is primarily driven by aid-based principles aimed at improving access to vaccines for vulnerable populations, with procurement and distribution facilitated through multilateral organizations and transnational public-private partnerships (TPPPs). As shown in Figure 3, the Coalition for Epidemic Preparedness Innovations (CEPI) supports vaccine R&D for epidemic threats (14); WHO oversees the Prequalification (PQ) programme to ensure quality standards for UN procurement (15); UNICEF serves as the largest single vaccine buyer within the UN system (16, p. 8); GAVI finances and coordinates vaccine delivery in eligible countries (17); the Pan American Health Organization (PAHO) facilitates regional procurement through its Revolving Fund (18). Overall, the key components of the global vaccine aid market are summarized in Table 1.

**Table 1:** Key components of the global vaccine aid market.

Key component	Description
Eligibility	Targets countries with limited purchasing power or insufficient infrastructure to procure vaccines independently.
Financing	Primarily funded by international organizations which in turn receive financing from donor governments and private foundations
Procurement channels	Vaccines are procured through multilateral mechanisms such as the GAVI-financed UNICEF procurement system, the PAHO Revolving Fund, and the WHO Prequalification (PQ) programme to ensure quality for UN procurement.
Governance mechanisms	Involves coordination among international organizations (e.g., WHO, UNICEF, PAHO) and TPPPs like CEPI, which de-risks vaccine R&D, and GAVI, which supports large-scale and low-cost procurement and delivery.

Source: Authors’ table. Factual elements drawn from CEPI vaccine R&D support (Coalition for Epidemic Preparedness Innovations, Accessed July 22, 2025), WHO Prequalification programme (World Health Organization, Accessed July 22, 2025), UNICEF procurement data (United Nations Children’s Fund, Accessed July 22, 2025), GAVI financing and delivery (Gavi, the Vaccine Alliance, Accessed July 22, 2025), and PAHO Revolving Fund operations (Pan American Health Organization, Accessed July 22, 2025).

Among these international actors, CEPI and GAVI are particularly noteworthy as they operate as TPPPs, distinct from the UN system agencies (19). Despite sharing a similar institutional form, they differ in functional orientation: CEPI focuses on upstream intervention by de-risking vaccine innovation through early-stage R&D funding, while GAVI operates downstream, shaping vaccine markets and supporting large-scale procurement and delivery in low-income countries. These coordinated mechanisms have significant market-shaping power: vaccines procured through UNICEF accounted for approximately 33% of global volumes (doses, calendar year 2023) (20), including 13% of total doses procured on behalf of GAVI for eligible countries (calendar year 2023) (20).

### 2.3 Analysis and synthesis

Overall, the global vaccine market is structurally bifurcated into self-procuring and donor-funded aid segments. In the self-procuring market, traditional market mechanisms operate: countries with sufficient purchasing power directly procure vaccines to meet national needs. Conversely, the aid segment is organized through a ‘super’ PPP model that integrates donor financing, regulatory oversight, pooled procurement, volume guarantees, and tiered pricing (21). This integrated structure demonstrates how coordinated mechanisms can shape markets and strategically direct resources to underserved populations. However, the model also presents significant challenges for equitable vaccine distribution, rendering the global aid market inherently complex.

During the COVID-19 pandemic, a small number of manufacturers concentrated in high-income countries prioritized the self-procuring market, thereby restricting supply to the aid segment and exposing critical weaknesses in mechanisms intended to ensure fair access (22). This tension between supply-side constraints and the normative objectives of global public health underscores the persistent vulnerability of aid-oriented mechanisms to market dynamics and highlights the urgent need for institutional reforms, including diversifying vaccine suppliers and strengthening local production capacity of recipient countries to enhance self-reliance, fairness, and accessibility.

## 3 The evolving role of Chinese enterprise in global vaccine aid market

Chinese enterprise’s integration into the global vaccine aid market unfolded in two distinct phases, with the COVID-19 pandemic marking a critical juncture.

### 3.1 Phase I (2011–2019)

Phase I (2011–2019) marked a period of gradual and limited integration of Chinese vaccine manufacturers into the global vaccine aid market. This stage was characterized by incremental regulatory breakthroughs, cautious entry into UN procurement systems, early international partnerships, and modest measurable outcomes. Prior to 2011, Chinese vaccines were largely excluded from international aid markets due to the absence of a WHO-recognized regulatory authority. This barrier was lifted in March 2011, when China’s National Regulatory Authority (NRA) was assessed by the WHO and deemed fully compliant with international standards, thereby granting Chinese-produced vaccines eligibility to apply for WHO PQ (23).

*New PQ entries*: Between 2013 and 2019, four Chinese-produced vaccines obtained WHO PQ—including the Japanese Encephalitis Vaccine Live (2013), Hualan influenza vaccine (2015), Sinopharm’s Poliomyelitis Vaccine (2017), and Sinovac’s HEALIVE (2017)—representing China’s initial regulatory breakthrough (see Table 1).

*Entry into UN procurement*: Following PQ approval, Chinese vaccines entered UNICEF- and GAVI-coordinated procurement systems for the first time. The CD-JEV vaccine was procured through UNICEF and deployed in national immunization campaigns in Laos, Nepal, and Cambodia, where Japanese encephalitis remains endemic (24).

*Partnerships and capacity-building investments*: During this phase, PATH collaborated with the Chengdu Institute of Biological Products to support PQ dossier submission and adaptation of cold-chain standards, representing one of the earliest cases of Sino-international technical cooperation in vaccine production (25).

*Quantifiable outcomes*: By 2019, only four Chinese-produced vaccines had achieved WHO PQ, and while publicly disclosed UNICEF procurement and delivery volumes remain limited, precise data on the number of lots and total dose volumes are not fully available for verification.

### 3.2 Phase II (2020—present)

Phase II (2020–present) is characterized by deepened participation of Chinese vaccine manufacturers, accelerated regulatory recognition, broader engagement in UN procurement, strengthened international partnerships, and record-setting quantitative outcomes, driven by the COVID-19 pandemic and China’s strategic commitment to global health cooperation.

*New PQ/EUL entries*: As shown in Table 2, since 2020, six Chinese-made vaccines have obtained WHO Emergency Use Listing (EUL) or PQ—including Beijing Bio-Institute of Biological Products (Sinopharm)‘s SARS-CoV-2 Vaccine (EUL, May 2021), Sinovac’s COVID-19 Vaccine (EUL, June 2021), CanSino’s Ad5-nCoV/Convidecia (EUL, May 2022), Innovax’s HPV vaccine Cecolin^®^ (PQ, Oct 2021), and Sinovac’s Poliomyelitis Vaccine and Varicella vaccines (PQ, June and Nov 2022). This represents a substantial expansion of China’s globally recognized vaccine portfolio.

**Table 2:** Timeline of Chinese vaccines with WHO prequalification (PQ) and emergency use listing (EUL).

Date	Vaccine name	Manufacturer	Status (PQ/EUL)
Phase I			
Oct 2013	Japanese Encephalitis Vaccine Live (SA14-14-2)	Chengdu Institute of Biological Products	PQ
June 2015	Seasonal Influenza Vaccine	Hualan Biological Engineering	PQ
Dec 2017	Poliomyelitis Vaccine (live, oral attenuated, human Diploid Cell)	Beijing Bio-Institute of Biological Products (Sinopharm)	PQ
Dec 2017	HEALIVE	Sinovac Biotech	PQ
Phase II			
May 2021	SARS-CoV-2 Vaccine (Vero Cell)	Beijing Bio-Institute of Biological Products (Sinopharm)	EUL
Jun 2021	COVID-19 Vaccine (Vero Cell)	Sinovac Biotech	EUL
Oct 2021	Bivalent HPV Vaccine (Cecolin^®^)	Xiamen Innovax Biotech	PQ
May 2022	Ad5-nCoV/Convidecia	CanSino Biologics	EUL
Jun 2022	Poliomyelitis Vaccine (Vero Cell)	Sinovac Biotech	PQ
Nov 2022	Varicella Vaccine (Live)	Sinovac (Dalian) Biotech	PQ

Source: WHO prequalification (PQ) and emergency use listing (EUL) databases; official WHO vaccine notices. URLs and access dates for each entry are provided in Supplementary File S2.

*Entry into UN procurement*: In 2021, GAVI and UNICEF signed supply agreements with Sinopharm (up to 170 million doses) and Sinovac (up to 380 million doses) for COVAX distribution to low- and middle-income countries (26). In parallel, UNICEF entered into procurement agreements for up to 120 million doses from Sinopharm and 200 million doses from Sinovac by the end of 2021 (27).

*Partnerships and capacity-building investments*: Chinese enterprises also deepened cooperation with global partners. In 2020, CEPI committed up to US $328 million to Clover Biopharmaceuticals to support the development, regulatory approval, and large-scale production of its protein-based S-Trimer COVID-19 vaccine candidate (28). Similarly, Shanghai Zerun Biotech and Walvax Biotech received up to US $25.1 million in 2022 to support preclinical and early clinical development of a COVID-19 variant vaccine, including Phase I trials and process optimization (29). These partnerships enhanced Chinese firms’ integration into the global vaccine R&D ecosystem. Moreover, Chinese producers established joint manufacturing facilities in the UAE, Serbia, Morocco, Brazil, Egypt, South Africa, and Chile, forming a network of more than 10 overseas vaccine production hubs aligned with the Belt and Road Initiative (BRI) (30).

*Quantifiable outcomes*: During the pandemic, China had commercially exported approximately 1.1 billion doses (calendar year 2021) of its COVID-19 vaccines to 123 countries. Of these, roughly 110 million doses (calendar year 2021) were procured by the COVAX global vaccine-sharing scheme to support vaccination in lower-income countries (31). In addition, China had delivered around 37 million doses (calendar year 2021) to the UN system as donations, out of a total pledged volume of approximately 52 million doses (31). Furthermore, Six Chinese vaccines were listed in WHO PQ/EUL databases, and Sinovac became the only Chinese firm with WHO-prequalified vaccines across multiple subsidiaries, reflecting a more institutionalized global presence (32, 33).

### 3.3 Analysis and synthesis

The longitudinal evolution of Chinese vaccine enterprises in the global aid market illustrates a trajectory of institutional learning, progressive internationalization, and adaptive strategic behavior. The transition from the first to the second phase signifies a structural transformation—from regulatory entry and cautious experimentation to deepen participation and partial normalization within the global health governance architecture. During Phase I, Chinese vaccine manufacturers adopted a deliberately cautious and risk-averse approach, navigating uncertainties related to international regulatory standards, competitive pressures from established suppliers, and reputational risks inherent in engaging with global health mechanisms. This incremental trajectory reflected a broader institutional logic in which Chinese enterprises prioritized regulatory compliance and the gradual accumulation of credibility over rapid market expansion. Their strategic orientation was closely aligned with the evolving legitimacy expectations of major global health actors such as WHO, UNICEF, and GAVI.

In contrast, Phase II illustrates both the rapid maturation and the enduring constraints of Chinese vaccine manufacturers’ engagement in the global aid market. On the one hand, the COVID-19 pandemic acted as a catalytic juncture, enabling Chinese firms to achieve unprecedented integration into WHO-led regulatory frameworks and UN procurement systems. This transition transformed them from peripheral participants into structural contributors to global immunization supply chains. On the other hand, persistent credibility gaps and geopolitical skepticism continued to delimit their international influence, underscoring the complex interplay between commercial globalization and normative legitimacy. In particular, doubts regarding the efficacy of Chinese COVID-19 vaccines—especially against the Delta and Omicron variants—provoked skepticism among Western media and international health actors, prompting global health partnerships such as GAVI and the Global Fund to adopt more cautious procurement practices (34, 35). Overall, this phase represents a pivotal moment in the institutionalization of Chinese vaccine enterprises as globally embedded yet strategically constrained actors—a dynamic that will be explored in greater depth in the following sections.

## 4 Underlying motivations for Chinese vaccine enterprises’ participation

### 4.1 Driven by long-term strategic gains

Scholars researching the internationalisation strategies of emerging markets companies have argued that their investment decisions can often be explained better by how investments contribute to the capability-building process of the firm than by calculations of short-term returns (36). This holds true for Chinese vaccine manufacturers as well. On the one hand, entry into the global aid market—particularly through mechanisms such as the WHO PQ program—requires substantial time and financial investment (37). On the other hand, vaccines procured by GAVI and UNICEF are characterized by high volume and low price points, which constrain short-term profitability.

A key motivation for Chinese vaccine manufacturers’ engagement in the global aid market arises from mounting domestic market pressures. Intensifying competition and emerging signs of overcapacity have eroded profit margins in China’s vaccine sector. As illustrated in Table 3, several leading companies—including Chongqing Zhifei Biological Products Co., Ltd.—have faced substantial declines in revenue and profit. Against this backdrop, many forward-looking firms have identified significant unmet vaccine demand in LMICs, particularly in Africa and in numerous BRI partner nations. While the domestic market becomes increasingly saturated, these regions face persistent shortages in both vaccine variety and supply. For Chinese manufacturers, securing WHO PQ functions as a critical gateway to these markets, viewed not merely as a regulatory hurdle but as a “strategic investment” in building and sustaining a presence in the Global South (38). As Meng Weining noted in an interview, WHO PQ is “one of the most important tools for going global—effectively, a passport to international markets” (39). An executive from another leading Chinese firm echoed this sentiment: “We have invested heavily in the PQ process, but we believe it holds long-term promise” (37).

**Table 3:** Performance overview of selected domestic vaccine companies.

Company name	Revenue 2024	Revenue 2023	YoY change (revenue)	Net profit 2024	Net profit 2023	YoY change (profit)
Chongqing Zhifei Biological Products Co., Ltd.	260.7	529.18	−50.74%	20.18	80.7	−74.99%
Watson Bio Co., Ltd.	28.21	41.14	−31.41%	1.42	4.19	−66.10%
CanSino Biologics Inc.	26.52	34.77	−23.75%	2.02	8.61	−76.59%
Wantai Biological Pharmacy Enterprise Co., Ltd.	22.45	55.11	−59.25%	1.06	12.48	−91.49%
Sichuan Biokin Pharmaceutical Co., Ltd.	12.29	18.25	−32.64%	2.32	5.01	−53.67%
Hualan Biological Vaccine Inc.	11.28	24.1	−53.21%	2.06	8.6	−76.10%
Beijing Jindike Biotech Institute Ltd.	0.81	1.35	−39.96%	−0.94	−0.71	−31.71%

Source: 21st Century Business Herald. Decoding the 2024 Performance of Vaccine Companies: Stuck in the “Price War” Quagmire, with “Innovation” and “Global Expansion” as Key Themes. 28 April 2025. Available online at: https://finance.sina.com.cn/roll/2025-04-28/doc-ineutqhs1813687.shtml?froms=ggmp (Accessed July 10, 2025) [Original text in Chinese].

A further motivation relates to the potential of vaccine manufacturing as a springboard for broader biopharmaceutical capacity-building in partner countries. Given that vaccine production is among the most technically demanding domains within the pharmaceutical sector, facilitating local manufacturing in BRI partner nations serves not only to address immediate public health needs but also to establish the foundation for sustainable capabilities in producing other essential medicines (30). This strategic approach allows Chinese firms to position themselves as long-term partners in strengthening healthcare systems abroad.

Finally, participation in the global aid market offers reputational advantages by enhancing brand recognition and reinforcing the image of Chinese vaccine companies as producers of high-quality, socially responsible products. For example, at the 2020 Global Vaccine Summit, Li Yunchun, Chairman of Watson Bio—the developer of a WHO-prequalified bivalent HPV vaccine—pledged to “continuously provide safe, effective, and affordable vaccines to populations worldwide, particularly in LMICs.” This commitment underscores the company’s dedication to promoting global public health by improving equitable access to vaccines through its active involvement in the global aid market (40). Similarly, regarding its commitment to building a reputation for high-quality vaccines, Sinovac CEO Yin Weidong emphasized: “Expanding beyond national borders to provide Chinese vaccines to children worldwide not only advances global health but also marks a key milestone—ensuring that children in China receive vaccines that meet the highest international quality standards” (41).

### 4.2 Incentives from state support

Chinese vaccine manufacturers’ entry into the global aid market has been strongly shaped by state support and top-down policy orientation, a trend particularly evident during the COVID-19 pandemic. Even before the crisis, Beijing had already laid the legislative groundwork for vaccine internationalisation. The Vaccine Administration Law, which came into effect in June 2019, explicitly encouraged manufacturers to “produce and export vaccines in line with international procurement requirements,” thus providing both legal endorsement and strategic guidance for aligning domestic production with global standards (42).

The onset of COVID-19 served as a powerful catalyst, accelerating the state’s integration of national vaccine producers into the global aid architecture—an effort closely linked to the Health Silk Road (HSR) initiative and the broader vision of building “a community of health for mankind” (10). At the 73rd World Health Assembly, President Xi Jinping pledged that Chinese vaccines would be made available as global public goods, signaling a strategic commitment to strengthening China’s role in the global aid market (43). To translate this pledge into action, the government rapidly mobilized national resources to accelerate vaccine development by domestic firms. On 21 January 2020, following high-level directives, the Ministry of Science and Technology established an inter-ministerial task force—bringing together the health, finance, education, and planning authorities—to coordinate the national COVID-19 vaccine R&D effort (44).

State-owned enterprises responded promptly to this mobilisation. Sinopharm explicitly framed itself as both a “state-owned enterprise” and a “key pillar” in advancing COVID-19 vaccine development and securing WHO emergency use authorization (EUA) (45). The company formed a dedicated team to prepare the Common Technical Document (CTD) required for WHO review and maintained continuous technical exchanges with WHO experts throughout 2020. This sustained engagement culminated in the December 2020 submission of the CTD and, following 4 months of review—including site inspections and iterative feedback—WHO granted EUA to Sinopharm’s inactivated COVID-19 vaccine, marking the first inclusion of a Chinese vaccine on the WHO EUL (46).

Government support was equally decisive in enabling Chinese vaccines to enter the procurement stage of the global aid market. In October 2020, Foreign Ministry spokesperson Hua Chunying announced China’s official participation in the COVAX Facility, describing it as “an important step to uphold the vision of a community of health for mankind and fulfill China’s commitment to making vaccines a global public good (47).” By July 2021, both Sinopharm and Sinovac vaccines were included in the COVAX portfolio through formal supply agreements with GAVI. According to China’s Permanent Mission to the United Nations Office at Geneva, this milestone would not have been achieved without “active support and encouragement” from the central government (48).

Importantly, this policy momentum has not subsided with the pandemic’s end. What began as an emergency coordination mechanism has gradually evolved into more institutionalised and locally targeted measures. For example, in May 2025, the Shanghai Municipal Government issued the *Opinions on Promoting the High-Quality Development of the Biomedical Industry,* introducing concrete incentives to support the internationalization of medical firms. Under this policy, innovative drugs and high-end medical devices developed in Shanghai that obtain registration from the WHO, the U.S. Food and Drug Administration (FDA), or the European Medicines Agency (EMA) are eligible for a one-time subsidy of up to 30% of their R&D investment, capped at 10 million RMB (49). Such measures suggest that the alignment of domestic industrial policy with global health ambitions is now becoming a sustained feature of China’s vaccine sector development strategy.

### 4.3 Analysis and synthesis

For enterprises from Global South countries, entering the global health governance arena poses a considerable latecomer disadvantage due to the longstanding monopolization by established Global North companies (39). Without a clear long-term strategic vision and robust national policy support, it is challenging for these firms to successfully expand internationally and participate effectively in the global aid market. Thus, the evolution of Chinese vaccine enterprises’ participation in the global aid market reveals a hybrid logic of strategic internationalization under state guidance. Unlike purely market-driven expansion, their engagement combines firm-level capability accumulation with state-led policy orchestration.

At the firm level, Chinese vaccine manufacturers’ strategic rationale extends well beyond short-term financial gains, encompassing long-term objectives such as regulatory compliance, global market access, manufacturing scale-up, and the cultivation of international brand reputation. Engagement with the WHO PQ program and UN procurement mechanisms functions not merely as a market-entry strategy but also as a structured pathway for institutional learning and legitimacy-building. By internalizing global regulatory standards, Chinese firms have enhanced their credibility, following an “institutional upgrading” trajectory analogous to that observed among latecomer firms in other high-standard, highly regulated industries. Consequently, their outward expansion can be interpreted as an adaptive response to both domestic market saturation and pressures to gain international recognition.

At the state level, Beijing’s active involvement transformed what might have remained firm-specific behavior into a nationally coordinated strategy. Through legislative endorsement, inter-ministerial coordination, and international diplomacy, Beijing lowered the transaction and coordination costs associated with global entry, embedding private and state-owned enterprises within a broader “Health Silk Road” narrative. This top-down coordination mechanism effectively integrated government agencies, research institutes, healthcare providers, and private firms to expedite all critical stages—from viral isolation and preclinical research to clinical trials—within compressed timeframes. According to Sinovac CEO Yin Weidong, this centralized task force provided critical financial and administrative support in expediting the company’s vaccine development (50). As he later observed, “Enterprises must remain attuned to national priorities and strive to meet the country’s needs.” (51).

This dual dynamic—bottom-up capability building and top-down political facilitation—has gradually positioned Chinese vaccine manufacturers as semi-autonomous agents within global health governance: commercially motivated yet aligned with public health objectives. However, this model also entails structural constraints. Reliance on state coordination may limit corporate autonomy and innovation flexibility, while persistent credibility gaps in Western-dominated regulatory regimes continue to circumscribe global influence. Accordingly, the Chinese experience illustrates both the opportunities and the boundaries of developmental internationalization in the context of global health markets.

## 5 Challenges for Chinese vaccine enterprises in the vaccine aid market

### 5.1 External challenges

Unlike their Western counterparts, Chinese vaccine manufacturers have faced a constellation of complex external barriers that have constrained their integration into the global vaccine aid market. These challenges have been compounded by the broader context of strategic competition between China and the United States, which has heightened the risk-averse posture of key global health actors. For example, insiders at the GAVI have indicated that, in the current geopolitical climate, the organization has often refrained from partnering with most Chinese pharmaceutical enterprises in order to “circumvent potential complications, such as the inclusion of Chinese companies on the U.S. Entity List” (52).

A further impediment stems from the latecomer disadvantage faced by companies from the Global South. Relative to established multinational corporations, Chinese vaccine firms possess less accumulated research capacity, weaker international reputations, and smaller-scale manufacturing capabilities—lagging not only behind firms from developed countries, but also behind traditional emerging-market producers such as India. As noted by senior executives of leading Chinese vaccine manufacturers, the CEPI tends to “prefer large multinational pharmaceutical companies” and remains “unfamiliar with and distrustful of Chinese firms” (53). Quantitative evidence supports these observations. Among the 25 companies funded by CEPI since its establishment, the vast majority—around 90 percent—are based in Western countries, with 9 located in the United States alone (54).

In the production phase, Indian companies account for nearly 60% of vaccines procured through UN agencies. Furthermore, UN procurement rules often cap the bid price of new entrants at or below that of incumbent suppliers, compressing profit margins for late entrants. This dynamic—low allowable prices combined with relatively higher production costs—limits both the capacity and the willingness of Chinese companies to participate in global vaccine supply. As one executive emphasized, “even if our primary goal is to open up the market rather than seek high profit margins, we must at least cover our costs to ensure survival” (53).

Finally, despite the expanding role of pharmaceutical firms from emerging markets such as China in supplying vaccines and contributing to R&D, their representation within the governance structures of the global aid market remains disproportionately limited, a situation that has drawn critical attention from scholars (36). In the case of GAVI, for instance, private philanthropic foundations and multinational pharmaceutical corporations exert substantial influence over agenda-setting and resource allocation. The Bill & Melinda Gates Foundation, a permanent board member alongside the WHO, UNICEF, and the World Bank, not only shapes GAVI’s internal priorities but also influences the broader global health policy landscape—often in alignment with the interests of large U.S.-based pharmaceutical companies (55). Empirical mapping of GAVI’s governance structure corroborates this pattern: of the 28 voting board members, 18 represent Northern countries, including governmental agencies, corporate actors, and public health experts from Western philanthropic organizations (56). Consequently, research institutions, publications, and policy forums in global health governance remain heavily concentrated in the United States, reflecting predominantly Northern-centric perspectives.

### 5.2 Internal challenges

For Chinese vaccine manufacturers, a limited willingness and lack of experience in engaging with the global aid market remain key barriers. Owing to relatively low levels of internationalization, many firms are unaware that such a market exists, and even those with some awareness often lack a clear understanding of the potential benefits. In particular, several manufacturers observed that large-scale procurement by UNICEF and GAVI could serve as a strategic gateway to broader market access, yet this opportunity remains underappreciated within the sector. One leading manufacturer acknowledged that, while they had been in contact with the WHO PQ process, they had “never once completed a full PQ cycle” (57). Another senior executive, when discussing potential participation in COVAX, candidly remarked that they were “still unclear about the process” and had “been waiting for clearer government directives, relying on anticipated fiscal support from public authorities” (53).

A shortage of internationally oriented talent further constrains participation. For many China’s manufacturers, the absence of a dedicated internationalization team—particularly one with both domain expertise in biopharmaceuticals and strong project management and cross-cultural communication skills—represents a critical bottleneck (58). Competing effectively in global vaccine markets requires mastery of international business norms, procurement procedures, and cross-border regulatory requirements, as well as the ability to cultivate a professional workforce adept at cross-cultural management.

A weak commitment to brand management also limits competitiveness. For multinational pharmaceutical corporations, brand-building is a core strategic asset that reinforces market recognition and consumer trust. By contrast, Chinese vaccine firms often lack a coherent branding strategy for export products, resulting in fragmented product planning and limited international visibility. This brand deficit is further exacerbated by reputational setbacks linked to domestic vaccine safety incidents, which have attracted global media scrutiny. For instance, a 2019 BBC report highlighted a vaccine safety crisis in Jiangsu province, where over a hundred children received expired vaccines, leading to multiple adverse reactions (59).

### 5.3 Analysis and synthesis

The challenges facing Chinese vaccine manufacturers in the global vaccine aid market reveal deeply entrenched structural asymmetries operating across both systemic and organizational levels. Externally, geopolitical rivalries, institutional biases, and latecomer disadvantages jointly constrain market access and partnership opportunities. Internally, limited internationalization capacity, weak brand management, and overreliance on state coordination have curtailed firms’ ability to act autonomously within global governance frameworks.

At the systemic level, the interplay of political risk and institutional path dependence has created an uneven playing field where emerging-market firms are assessed through the prism of global power asymmetries rather than purely technical criteria. Intensifying strategic competition between China and the United States has heightened the risk sensitivity of key intermediaries such as GAVI and CEPI, prompting cautious or exclusionary attitudes toward Chinese participation. This politicization of partnership governance transforms global health from a normatively neutral domain into a stratified arena in which access and legitimacy are filtered through geopolitical alignment.

A further layer of constraint stems from governance imbalances within major global health institutions. Despite increasing contributions from emerging-market producers, decision-making power remains concentrated among a small group of Western philanthropic and corporate actors. The underrepresentation of Chinese firms on governing bodies—illustrated by the fact that, aside from the appointment of Yibing Wu (CEO of Temasek China) as an independent board member, no senior executives from Chinese vaccine producers have served on GAVI’s Board—perpetuates informational asymmetries and constrains influence over agenda setting, procurement norms, and regulatory harmonization. As a result, Chinese manufacturers are incorporated into the global vaccine supply chain primarily as suppliers rather than as rule-shaping participants, reinforcing their peripheral position within the global vaccine governance architecture.

At the organizational level, internal weaknesses amplify these systemic asymmetries. Many Chinese firms remain reactive toward global aid mechanisms, awaiting state direction and fiscal incentives rather than proactively identifying strategic opportunities. A shortage of internationally experienced personnel and the absence of coherent brand and communication strategies further hinder their ability to navigate complex regulatory environments and cultivate global trust. Reputational vulnerabilities continue to erode credibility and slow the institutionalization of trust within international regimes.

Overall, the interaction between external geopolitical constraints and internal organizational limitations produces a dual structural challenge. China’s integration into the global vaccine aid market thus represents not merely a trajectory of industrial upgrading but a complex negotiation within the political economy of global health governance. While the developmental internationalization model has accelerated capacity building, its long-term success will depend on the ability of Chinese vaccine manufacturers to evolve from policy-driven implementers into norm entrepreneurs. These enterprises would then not only help shape the global health order but also actively promote vaccine equity across the developing world.

## 6 Conclusion and policy implications

Unlike existing studies that focus primarily on China’s macro-level role in global health governance, this study provides a novel and comprehensive analysis of the evolving role of Chinese vaccine manufacturers in the global vaccine aid market, highlighting the opportunities, motivations, and challenges that shape their participation. By analyzing the sector before and after the COVID-19 pandemic, it becomes evident that Chinese enterprises have gradually expanded their international presence, leveraging regulatory achievements, participation in UN procurement programs, and strategic alignment with initiatives such as the Health Silk Road and the Belt and Road Vaccine Partnership. These efforts reflect a dual motivation: advancing public health outcomes in LMICs while simultaneously enhancing corporate visibility, credibility, and long-term market access.

Despite these advances, significant challenges remain. Externally, geopolitical frictions, latecomer disadvantages, and limited representation in global governance structures continue to constrain engagement. Internally, inadequate internationalization experience, shortages of globally oriented talent, and weak brand management hinder firms’ capacity to fully integrate into the global health landscape. The COVID-19 pandemic underscored both the promise and the limitations of Chinese vaccine manufacturers’ participation, serving as an inflection point for reconsidering the role of emerging-market firms in global health governance.

By foregrounding corporate actors alongside state-level dynamics, this study offers new insights into China’s participation in global health governance and contributes to scholarship on global vaccine aid, public-private partnership models, North–South health disparities, and corporate behavior in international health governance. The findings also carry clear policy relevance, informing international institutions on how to engage emerging-market firms effectively and sustainably in equitable global health initiatives.

From a policy perspective, international institutions such as WHO, PATH, GAVI, CEPI, and UNICEF should recognize Chinese government’s increasing willingness to assume greater responsibilities in global health. Notably, Beijing pledged USD 500 million to the WHO in May 2025, signaling stronger political commitment at the state level (60). However, financial contributions alone are insufficient to enable China to play a comprehensive and long-term role in global health governance. Sustained progress requires the construction of a multi-layered engagement framework that aligns state-level policy leadership with the internationalization of enterprises, strengthens their technical and managerial capacity to meet global standards, and incorporates the broader support of civil society and professional networks.

To enhance feasibility and provide actionable guidance, international institutions such as WHO, UNICEF, GAVI, and CEPI should adopt a multi-stage approach that accounts for c constraints:

- Short term (1–2 years): Build trust and lower entry barriers

- For WHO PQ: Pilot staggered review pathways for late-entry manufacturers that meet predefined comparability criteria, enabling technically capable Chinese firms to gain incremental access without delaying established producers. *Enabling conditions*: sufficient staffing and transparent criteria. *Risks*: perceptions of preferential treatment must be mitigated through public disclosure.
- For UNICEF/GAVI: Organize targeted technical mentoring, joint workshops, and pilot procurement schemes to familiarize Chinese firms with WHO PQ standards, UNICEF bidding systems, and GAVI supply chain protocols. *Enabling conditions*: collaboration with neutral technical partners (e.g., PATH). *Risks*: geopolitical tensions could affect participation if perceived as politically instrumentalized.

- Medium term (3–5 years): Strengthen managerial and operational capacities

- For CEPI/WHO: Establish dedicated technical assistance platforms to guide product registration, quality assurance, and compliance audits, enabling Chinese firms to participate in co-development and certification partnerships. *Enabling conditions*: sustained funding and expert engagement. *Risks*: capacity constraints or bureaucratic delays.
- For GAVI/UNICEF: Incentivize consortium-based procurement to gradually diversify the global supply base beyond incumbent multinational firms. *Enabling conditions*: transparent allocation rules. *Risks*: resistance from entrenched suppliers or governments favoring established partners.

- Long term (beyond 5 years): Align national and enterprise-level frameworks

- For all institutions: Facilitate institutional alignment through sustained public-private dialogues, harmonized regulatory pathways, and regional vaccine hubs integrating Chinese and local producers in LMICs. *Enabling conditions*: political will and multi-stakeholder commitment. *Risks*: mutual distrust, competition with entrenched suppliers, and resistance from governance structures dominated by established Western actors.
- For LMIC agencies and local partners: Develop template memoranda of understanding (MOUs) for technology transfer that include milestone-based performance payments. This ensures accountability, incentivizes timely capacity building, and facilitates integration of Chinese producers into regional manufacturing networks. *Enabling conditions*: supportive legal frameworks and monitoring mechanisms. *Risks*: political or institutional resistance.

Such a staged approach allows international institutions to deepen Chinese vaccine manufacturers’ understanding of the operational and strategic benefits of participating in the global vaccine aid market, promoting sustainable integration, resilient supply chains, and equitable vaccine access. By leveraging cross-subsidy mechanisms, the structural and operational capacities developed through these staged interventions can be translated into practical outcomes—channeling revenues from wealthier markets to support vaccine provision in lower-income regions, thereby reinforcing both market sustainability and equitable access. At the same time, the implementation of this approach should be guided by ethical considerations to ensure that the pursuit of efficiency and inclusiveness does not compromise the principles of transparency, accountability, and equity. International institutions and China’s corporate partners must remain vigilant against potential unintended consequences—such as the instrumentalization of vaccine aid for geopolitical purposes or the marginalization of local producers in recipient countries.

Closer partnerships between international institutions and Chinese enterprises could create mutual benefits: global health organizations would gain access to China’s financial and technological resources, thereby diversifying supply chains and reducing dependence on a small pool of multinational vaccine producers; meanwhile, Chinese vaccine firms would acquire valuable exposure to international standards, procurement mechanisms, and governance practices, accelerating their integration into the global health system. The experience of COVID-19 has made clear that concentrating vaccine R&D and production in a limited number of firms cannot meet global demand during crises. Harnessing the capacities of vaccine enterprises in emerging economies like China can strengthen the resilience of global vaccine supply chains, diversify sources, and advance equitable access, even in the face of resource and institutional constraints.

Finally, it should be emphasized that, while the study identifies clear trends and policy-relevant insights, several methodological constraints should be noted. This analysis relies primarily on English-language sources, with Chinese-language materials used as supplementary references. First, dependence on publicly available sources may introduce language and publication biases. Second, potential selection bias exists in interview-based data, as participants may not fully represent the range of perspectives from Chinese or international stakeholders. Third, some proprietary procurement and pricing data could not be independently verified, which may limit the precision of quantitative estimates. Acknowledging these limitations helps contextualize the findings and highlights avenues for future research, such as incorporating multilingual sources, broader stakeholder engagement, and access to proprietary data.
