# Supplementary material for: A critical review of Chinese vaccine enterprises in the global aid market: evolution, drivers, and structural constraints
Source: Front Public Health. 2025 Dec 5;13:1692140. doi: 10.3389/fpubh.2025.1692140 (PMC12714872; doi:10.3389/fpubh.2025.1692140)
Supplement: Supplementary file 1 [file Data_Sheet_1.docx]

| **Database** | **Search string** | **Date run** | **Records retrieved** | **Notes** |
| --- | --- | --- | --- | --- |
| Web of Science | (“China” OR “Chinese”) AND (“global health governance” OR “vaccine cooperation” OR “Health Silk Road”) | 2025-05-16 | 570 | Applied filters: English, 2015–2025 |
| PubMed | (“China” OR “Chinese”) AND (“global health governance” OR “vaccine cooperation” OR “Health Silk Road”) | 2025-05-16 | 1076 | Applied filters: English, 2015–2025 |

**Supplementary File S1. The Full Strategies of Boolean Operators and Truncations**

**Supplementary File S2. Codebook for Thematic Analysis**

| ****Code**** | ****Theme / Subtheme**** | ****Definition**** | ****Illustrative Example****  ****(from Interviews)**** |
| --- | --- | --- | --- |
| **C1** | **State-led coordination in global health governance** | References to China’s governmental leadership in structuring or financing international vaccine cooperation. | “Multiple government agencies jointly led the alignment of enterprise participation with the Health Silk Road agenda.” |
| **C2** | **Public–private collaboration mechanisms** | Descriptions of coordination or tensions between government agencies, enterprises, and international partners. | “Our partnership with Gavi required navigating both commercial contracts and diplomatic issues.” |
| **C3** | **Strategic motivations and diplomatic framing** | Statements linking enterprises’ vaccine cooperation to broader foreign policy or health diplomacy objectives. | “The vaccine initiative was part of China’s effort to demonstrate responsible global leadership.” |
| **C4** | **Institutional and regulatory constraints** | Mentions of bureaucratic hurdles, international trust deficits, or quality certification challenges of Chinese vaccine companies. | “Regulatory approval delays limited the speed of China’s vaccine exports.” |
| **C5** | **Market access and competitive positioning** | Discussions of how Chinese enterprises engage with global vaccine markets, donors, and procurement agencies. | “Unlike Western firms, we rely heavily on bilateral agreements for overseas distribution.” |
| **C6** | **Knowledge transfer and capacity building** | Insights into technology sharing, local production partnerships, or training programs. | “Technology transfer in Africa was critical to improving local vaccine self-sufficiency.” |
| **C7** | **Normative perceptions and reputational concerns** | Expressions of how Chinese enterprise’s role and credibility are perceived internationally. | “Despite the donations, some partners still questioned the transparency of vaccine data.” |
| **C8** | **Evolution of the Health Silk Road (HSR)** | References to how the HSR framework adapted to pandemic-related challenges or global criticism. | “After COVID-19, the HSR emphasized health security and supply chain resilience.” |

**Supplementary File S3. Semi-Structured Interview Guide**

**Purpose:**

To explore how China’s state actors, enterprises, and international partners perceive and operationalize Chinese firms’ participation in global aid vaccine market within the framework of global health governance.

**Section 1. Background and Role**

Can you describe your organization’s role in China’s participation in global aid vaccine market?

How has your organization’s mandate or operational role changed since 2015, especially with the shift from domestic production capacity building to international market engagement?

**Section 2. National Strategy, Policy Design, and Inter-Agency Coordination**

How are national strategies (e.g. the Health Silk Road, vaccine as a “global public good”) translated into concrete instruments (funding instruments, supply agreements, standard-setting, etc.)?

What coordination mechanisms (formal or informal) exist among ministries, regulatory bodies, and SOEs/private vaccine enterprises to align external signalling and actual delivery?

What are the main challenges in policy coherence or inter-agency coordination?

**Section 3. Enterprise-Level Implementation**

How do Chinese vaccine enterprises participate in international procurement or aid mechanisms (e.g., COVAX, Gavi)?

What are the key incentives and constraints shaping enterprise participation?

To what extent do Chinese firms perceive this market as commercial, strategic, or norm-embedding?

**Section 4. Partnerships and International Perceptions**

How would you assess Chinese firms’ interactions with Gavi, WHO, CEPI, UNICEF, etc. during COVID-19 vaccine deployment?

How do international partners evaluate China’s quality standards, regulatory maturity, supply reliability, and contractual behaviour?

Do you observe persistent biases or epistemic stereotypes in how Chinese vaccine suppliers are perceived?

**Section 5. Institutional Lessons and Forward Trajectories**

What governance or institutional lessons emerged from Chinese firm’s involvement in the global aid vaccine market?

If Chinese firms seek a more institutionalized or rule-shaping role in global health governance — what are the most realistic pathways in the next 5–10 years?

**Supplementary File S4. WHO PQ/EUL official sources and URLs**

| **Vaccine Name** | **WHO PQ/EUL Link** | **Access Date** |
| --- | --- | --- |
| Japanese Encephalitis Vaccine Live (SA14-14-2) | https://extranet.who.int/prequal/vaccines/p/japanese-encephalitis-vaccine-live-sa14-14-2 | 10 May 2025 |
| Seasonal Influenza Vaccine | https://www.who.int/hongkongchina/news/detail/12-06-2015-who-prequalifies-chinese-influenza-vaccine | 10 May 2025 |
| Poliomyelitis Vaccine (live, oral attenuated, human Diploid Cell) | https://extranet.who.int/prequal/vaccines/p/poliomyelitis-vaccine-live-oral-attenuated-human-diploid-cell-type-1-and-3 | 10 May 2025 |
| HEALIVE | https://extranet.who.int/prequal/vaccines/p/healive | 10 May 2025 |
| SARS-CoV-2 Vaccine (Vero Cell) | https://extranet.who.int/prequal/sites/default/files/document_files/Summary%20Status%20of%20COVID-19%20Vaccines%20within%20WHO%20EUL-PQ%20evaluation%20process%20-%2001%20July%202025%20.pdf | 10 May 2025 |
| COVID-19 Vaccine (Vero Cell) | https://extranet.who.int/prequal/sites/default/files/document_files/Summary%20Status%20of%20COVID-19%20Vaccines%20within%20WHO%20EUL-PQ%20evaluation%20process%20-%2001%20July%202025%20.pdf | 10 May 2025 |
| Bivalent HPV Vaccine (Cecolin®) | https://extranet.who.int/prequal/vaccines/p/cecolinr | 10 May 2025 |
| Ad5-nCoV/Convidecia | https://extranet.who.int/prequal/sites/default/files/document_files/Summary%20Status%20of%20COVID-19%20Vaccines%20within%20WHO%20EUL-PQ%20evaluation%20process%20-%2001%20July%202025%20.pdf | 10 May 2025 |
| Poliomyelitis Vaccine (Vero Cell) | https://extranet.who.int/prequal/vaccines/p/poliomyelitis-vaccine-vero-cell-inactivated-sabin-strains | 10 May 2025 |
| Varicella Vaccine (Live, attenuated) | https://extranet.who.int/prequal/vaccines/p/varicella-vaccine-live | 10 May 2025 |

**Supplementary File S5. Claim–Evidence Map with Conceptual Labels**

| **No.** | **Claim / Normative Statement** | **Conceptual Label** | **Type of Evidence** | **Source / Citation** |
| --- | --- | --- | --- | --- |
| 1 | GAVI has refrained from partnering with most Chinese pharmaceutical enterprises to “circumvent potential complications, such as the inclusion of Chinese companies on the U.S. Entity List.” | **Geopolitical constraint** | Interview-derived perception | Interview with senior executive of GAVI, May 2023 |
| 2 | CEPI tends to “prefer large multinational pharmaceutical companies” and remains “unfamiliar with and distrustful of Chinese firms.” | **Institutional mistrust** | Interview-derived perception | Interview with senior executive of a Chinese vaccine company, Aug 2020 |
| 3 | About 90% of CEPI-funded companies are based in Western countries, with 9 in the U.S. | **Structural asymmetry** | Official dataset | CEPI, Vaccine Technology (accessed 22 July 2025) |
| 4 | Indian companies account for nearly 60% of vaccines procured through UN agencies. | **Market concentration** | Official dataset | UNICEF/WHO vaccine market reports |
| 5 | UN procurement rules cap the bid price of new entrants at or below incumbents’, compressing profit margins. | **Institutional bias / Latecomer disadvantage** | Official rule + interview | UN Procurement Manual; interview (Aug 2020) |
| 6 | Philanthropic foundations and multinational corporations dominate GAVI’s agenda-setting and resource allocation. | **Governance imbalance** | Scholarly literature | Harman, S. (2016). Global Governance, 22(3), 349–368 |
| 7 | Of GAVI’s 28 voting members, 18 represent Northern actors. | **Governance imbalance / North–South asymmetry** | Official document | GAVI Board webpage (accessed 22 July 2025) |
| 8 | Research and policy forums in global health governance remain concentrated in the U.S., reflecting Northern-centric perspectives. | **Epistemic dominance** | Scholarly synthesis | Roemer-Mahler, A. (2014). Review of International Studies, 40, 897–918 |
| 9 | Many Chinese manufacturers remain reactive, awaiting state direction rather than proactively engaging. | **Organizational dependence** | Interview-derived perception | Interviews with Chinese vaccine executives (Aug 2020); officials (May 2025) |
| 10 | Reputational vulnerabilities stemming from domestic vaccine safety incidents undermine global credibility. | **Reputational vulnerability** | Media report | BBC (2019). China vaccine scandal: Over 100 Jiangsu children given expired vaccines |
| 11 | Politicization of partnership governance transforms global health from a neutral to a stratified arena. | **Geopolitical politicization** | Analytical inference (mixed evidence) | Synthesized from GAVI/CEPI governance docs + interviews |
| 12 | Chinese vaccine firms are integrated as suppliers rather than rule-shaping participants in global governance. | **Structural marginalization** | Documented fact + interpretation | GAVI Board records; author synthesis |
| 13 | A shortage of internationally oriented talent limits global engagement capacity. | **Talent shortage / Organizational weakness** | Interview-derived perception | Interviews with government officials, Beijing, May 2025 |

**Note:** Conceptual labels (e.g., institutional mistrust, reputational vulnerability, geopolitical politicization) indicate interpretive categories that emerged from thematic coding of interview and document data. Statements marked as interview-derived perceptions represent the subjective views of respondents rather than verified institutional facts.
